# Supplementary material for: TGR5 signalling promotes mitochondrial fission and beige remodelling of white adipose tissue
Source: Nat Commun. 2018 Jan 16;9:245. doi: 10.1038/s41467-017-02068-0 (PMC5770450; doi:10.1038/s41467-017-02068-0)
Supplement: Supplementary file 1 — Supplementary Information [file 41467_2017_2068_MOESM1_ESM.pdf]

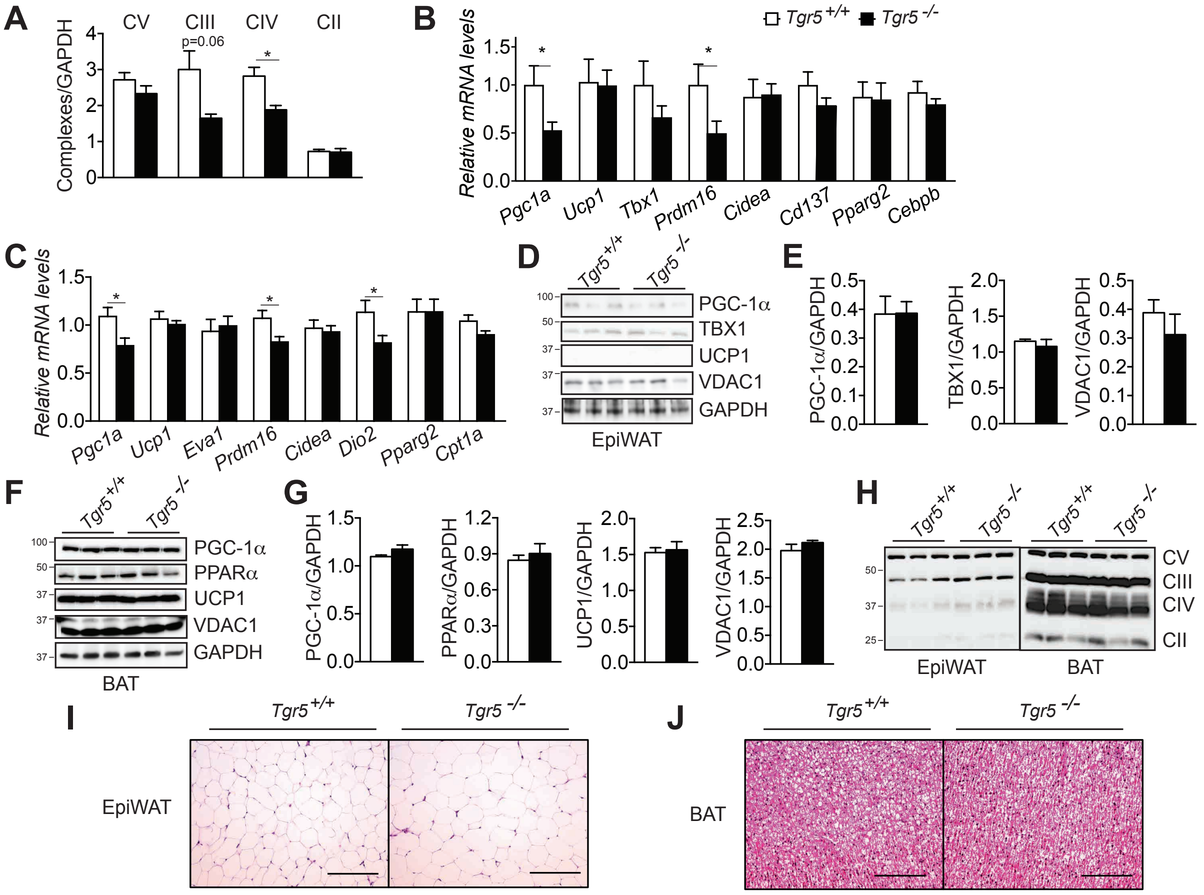

Supplementary Fig. 1. Analysis of beiging, mitochondrial and adipogenic markers in EpiWAT and BAT from *Tgr5*$^{+/+}$ and *Tgr5*$^{-/-}$ mice exposed to cold. (**A**) Quantitative densitometry of the Western blots depicted in **Figure 1h**. (**B** and **C**) mRNA expression levels of beige remodeling and mitochondrial markers *Pgc1a*, *Ucp1*, *Tbx1*, *Prdm16*, *Cidea*, *Cd137*, *Eva1*, *Dio2*, *Cpt1a, Pparg2* and *Cebpb* in the EpiWAT (**B**) and BAT (**C**) of TGR5 wild-type (*Tgr5*$^{+/+}$) and germline TGR5 knock-out (*Tgr5*$^{-/-}$) mice exposed to cold (8°C) for 7 days. *n* = 10 per group. (**D** and **F**) Representative (*n* = 10 per group) Western blot of PGC-1α, the mitochondrial marker VDAC1, and beiging markers TBX1 and UCP1 from the EpiWAT (**D**), and PGC-1α, PPARα, UCP1 and the mitochondrial marker VDAC1 from the BAT (**F**) of the mice described in **B** and **C**. GAPDH was used as loading control. (**E** and **G**) Quantitative densitometry of the Western blots showed in D (EpiWAT) and F (BAT). (**H**) Representative (*n* = 10 per group) Western blot of mitochondrial OXPHOS complexes (CII to CV) from the EpiWAT and BAT of the mice described in **B** and **C**. (**I** and **J**) Representative (*n* = 5 per group) hematoxylin and eosin stainings of EpiWAT (**I**) and BAT (**J**) sections from mice described in **A** and **B**. Scale bars = 50μm. Results represent mean ± SEM. * $P \leq 0.05$ vs. *Tgr5*$^{+/+}$ group by Student's t-test.

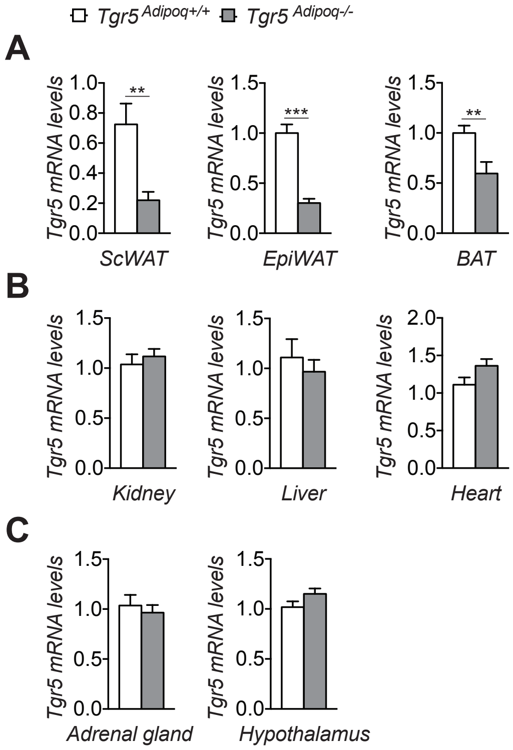

Supplementary Fig. 2. Validation of WAT specific TGR5 knock-out (*Tgr5*$^{Adipoq-/-}$) mice. (**A** to **C**) TGR5 mRNA expression levels in the indicated tissues of WAT specific TGR5 knock-out (*Tgr5*$^{Adipoq-/-}$) mice and their controls (*Tgr5*$^{Adipoq+/+}$) (n = 10 per group). Results represent mean ± SEM. ** $P \leq 0.01$ and *** $P \leq 0.001$ vs. *Tgr5*$^{Adipoq+/+}$ group by Student's t-test.

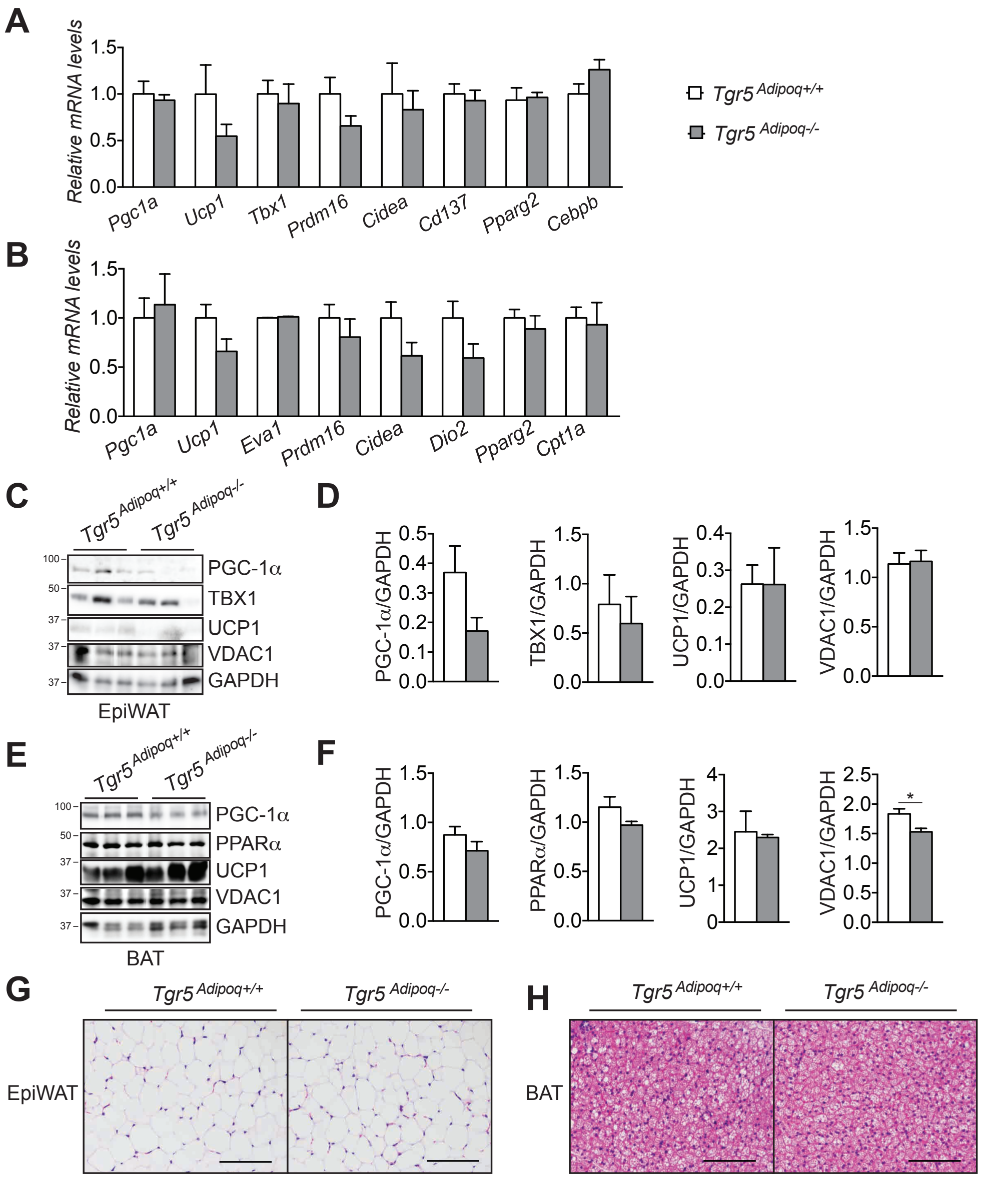

Supplementary Fig. 3. Analysis of beiging, mitochondrial and adipogenic markers in EpiWAT and BAT form *Tgr5*$^{Adipoq+/+}$ and *Tgr5*$^{Adipoq-/-}$ mice exposed to cold. (**A** and **B**) mRNA expression levels of beige remodeling and mitochondrial markers *Pgc1a*, *Ucp1*, *Tbx1*, *Prdm16*, *Cidea*, *Cd137*, *Eva1, Dio2*, *Cpt1a*, *Pparg2* and *Cebpb* in the EpiWAT (**A**) and BAT (**B**) of control mice (*Tgr5*$^{Adipoq+/+}$) and WAT specific TGR5 knock-out (*Tgr5*$^{Adipoq-/-}$) mice exposed to cold (8°C) for 7 days. *n* = 10 per group. (**C** and **E**) Representative (*n* = 10 per group) Western blot of PGC-1α, the mitochondrial marker VDAC1, and beiging markers TBX1 and UCP1 from the EpiWAT (**C**) and PGC-1α, PPARα, UCP1 and the mitochondrial marker VDAC1 from the BAT (**E**) of the mice described in **A** and **B**. GAPDH was used as loading control. (**D** and **F**) Quantitative densitometry of the Western blots showed in **C** (EpiWAT) and **E** (BAT). (**G** and **H**) Representative (*n* = 5 per group) hematoxylin and eosin stainings of EpiWAT (**G**) and BAT (**H**) sections from mice described in **A**. Scale bars = 50μm. Results represent mean ± SEM. * $P \leq 0.05$ vs. *Tgr5*$^{Adipoq+/+}$ group by Student's t-test.

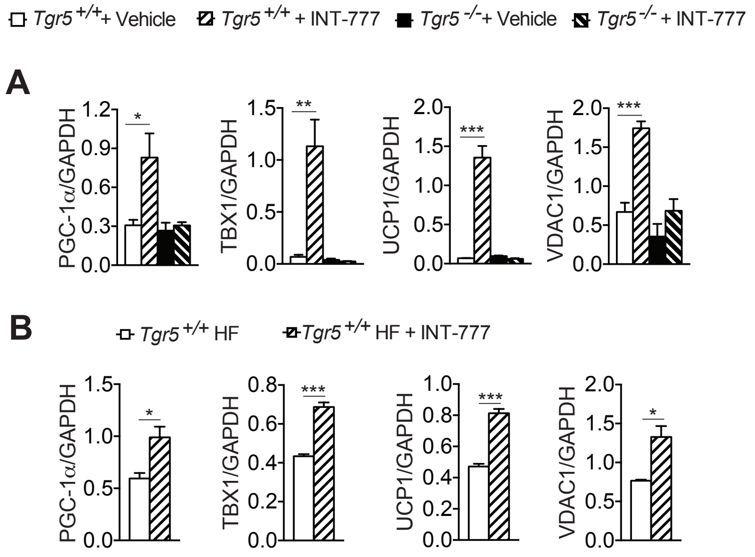

Supplementary Fig. 4. TGR5 activation promotes beiging in the scWAT at thermoneutrality and after high fat diet feeding. (**A**) Quantitative densitometry of the Western blots showed in **Figure 3e**. (B) Quantitative densitometry of the Western blots showed in **Figure 4c** (n = 10 per group). Results represent mean ± SEM. * $P \leq 0.05$, ** $P \leq 0.01$ and *** $P \leq 0.001$ vs. *Tgr5*$^{+/+}$ + Vehicle control group by one-way ANOVA followed by Bonferroni post-hoc test (**A**); or *Tgr5*$^{+/+}$ HF control group by Student's t-test (**B**).

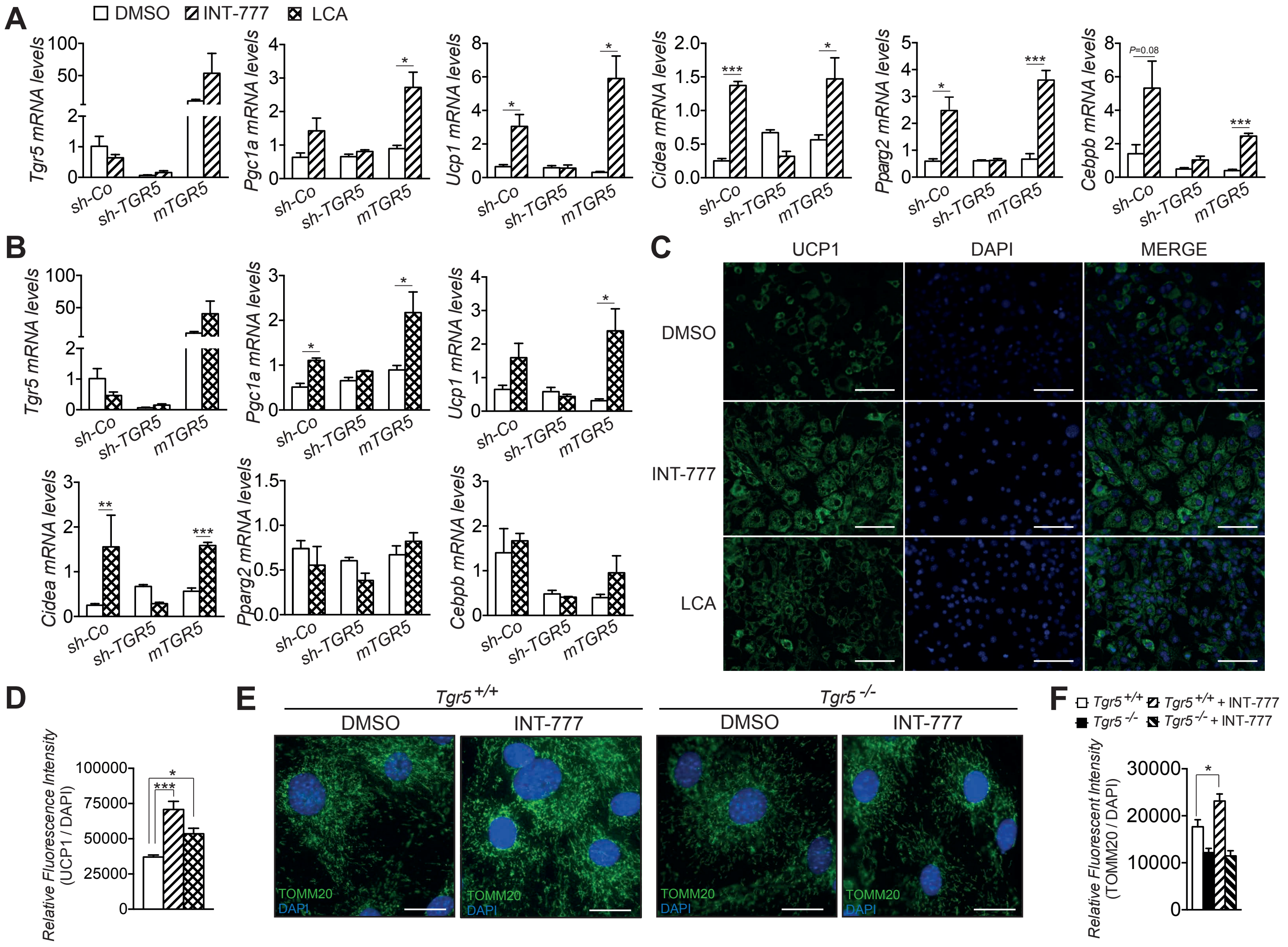

Supplementary Fig. 5. TGR5 activation promotes beige adipocyte differentiation *in vitro*. (**A** and **B**) mRNA expression levels of *Tgr5*, and beige remodeling markers *Pgc1a*, *Ucp1*, *Cidea*, *Pparg2* and *Cebpb* in differentiated 3T3-L1 cells in presence of the TGR5 agonists INT-777 (**A**), lithocholic acid (LCA) (**B**) or vehicle (DMSO). 3T3-L1 cells were transfected with either shRNA for TGR5 (sh-TGR5), control shRNA (sh-Co) or mouse TGR5 (mTGR5), as indicated in the Figure. *n* = 6 (**C**) Representative (*n* = 6 per group) images of UCP1 immunofluorescence (in green) on the cells described in **A** and **B**. Nuclei were stained with DAPI (in blue). Scale bars = 50μm. (**D**) Quantification of fluorescent intensity showed in **C**. Normalization of fluorescence was performed with cell count obtained by nuclei (DAPI) staining. **(E**) Representative (*n* = 6 per group) images of TOMM20 immunofluorescence (in green) of adipocytes differentiated from the stromal vascular fraction (SVF) of TGR5 wild-type (*Tgr5*$^{+/+}$) and germline TGR5 knock-out (*Tgr5*$^{-/-}$) mice. SVF cells were differentiated for 7 days in presence or absence of the TGR5 agonist INT-777. *n* = 6. Nuclei were stained with DAPI (in blue). Scale bars = 25μm (**F**) Quantification of fluorescent intensity showed in **E**. Results represent mean ± SEM. * $P \leq 0.05$, ** $P \leq 0.01$ and *** $P \leq 0.001$ vs. DMSO control group by Student's t-test (**A** and **B**), or by one-way ANOVA followed by Bonferroni post-hoc test (**D** and **F**).

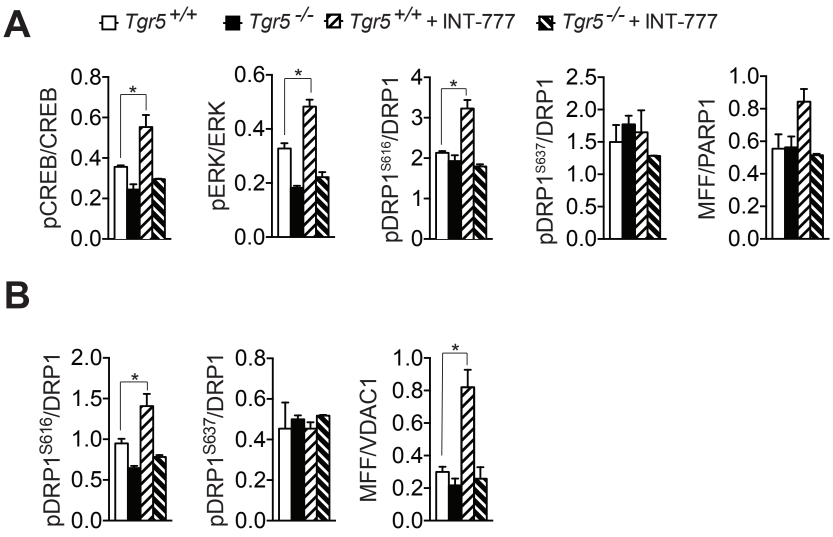

Supplementary Fig. 6. TGR5 activation induces mitochondrial fission. (**A**) Quantitative densitometry of the Western blots showed in **Figure 6e** (*n* = 6 per group). (**B**) Quantitative densitometry of the Western blots showed in **Figure 6f** (*n* = 6 per group). Results represent mean ± SEM. * $P \leq 0.05$, ** $P \leq 0.01$ and *** $P \leq 0.001$ vs. *Tgr5*$^{+/+}$ group by one-way ANOVA followed by Bonferroni post-hoc test.

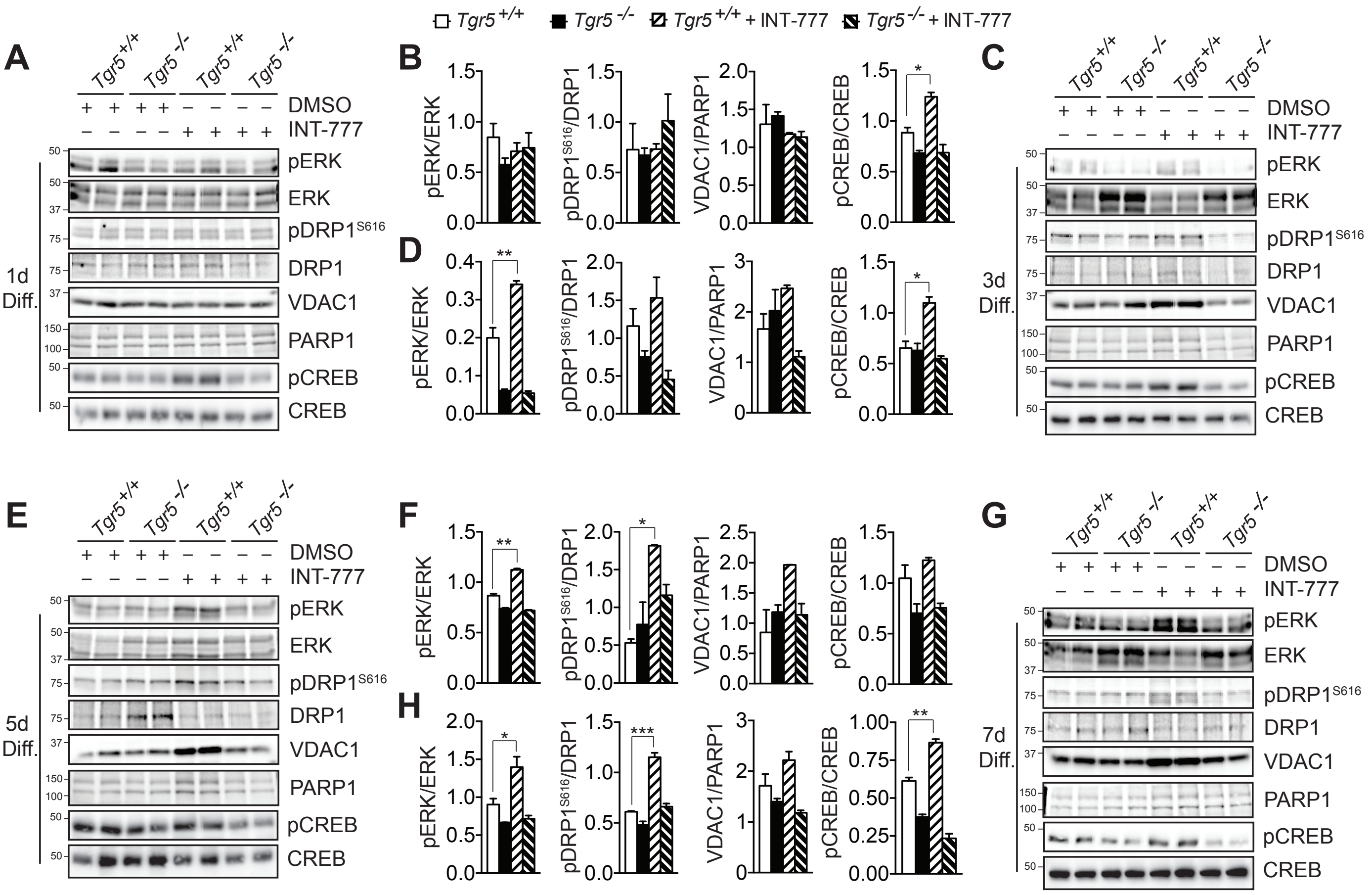

Supplementary Fig. 7. Effect of TGR5 activation on downstream signaling components during beige differentiation (**A**, **C**, **E** and **G**) Representative (n = 6 per group) Western blots of mitochondrial protein VDAC1 and TGR5 downstream targets (phospho proteins) with their relative controls (ERK, DRP1 or CREB) from differentiated adipocytes (different time points indicated in the figure) derived from the stromal vascular fraction (SVF) of TGR5 wild-type (*Tgr5*$^{+/+}$) and germline TGR5 knock-out (*Tgr5*$^{-/-}$) mice. PARP1 was used as a loading control. Cells were stimulated with the TGR5 agonist INT-777 or vehicle (DMSO). n = 6 (**B**) Quantitative densitometry of the Western blots showed in **A**. (**D**) Quantitative densitometry of the Western blots showed in **C**. (**F**) Quantitative densitometry of the Western blots showed in **E**. (**H**) Quantitative densitometry of the Western blots showed in **G**. Results represent mean ± SEM. * $P \leq 0.05$, ** $P \leq 0.01$ and *** $P \leq 0.001$ vs. *Tgr5*$^{+/+}$ DMSO group by one-way ANOVA followed by Bonferroni post-hoc test.

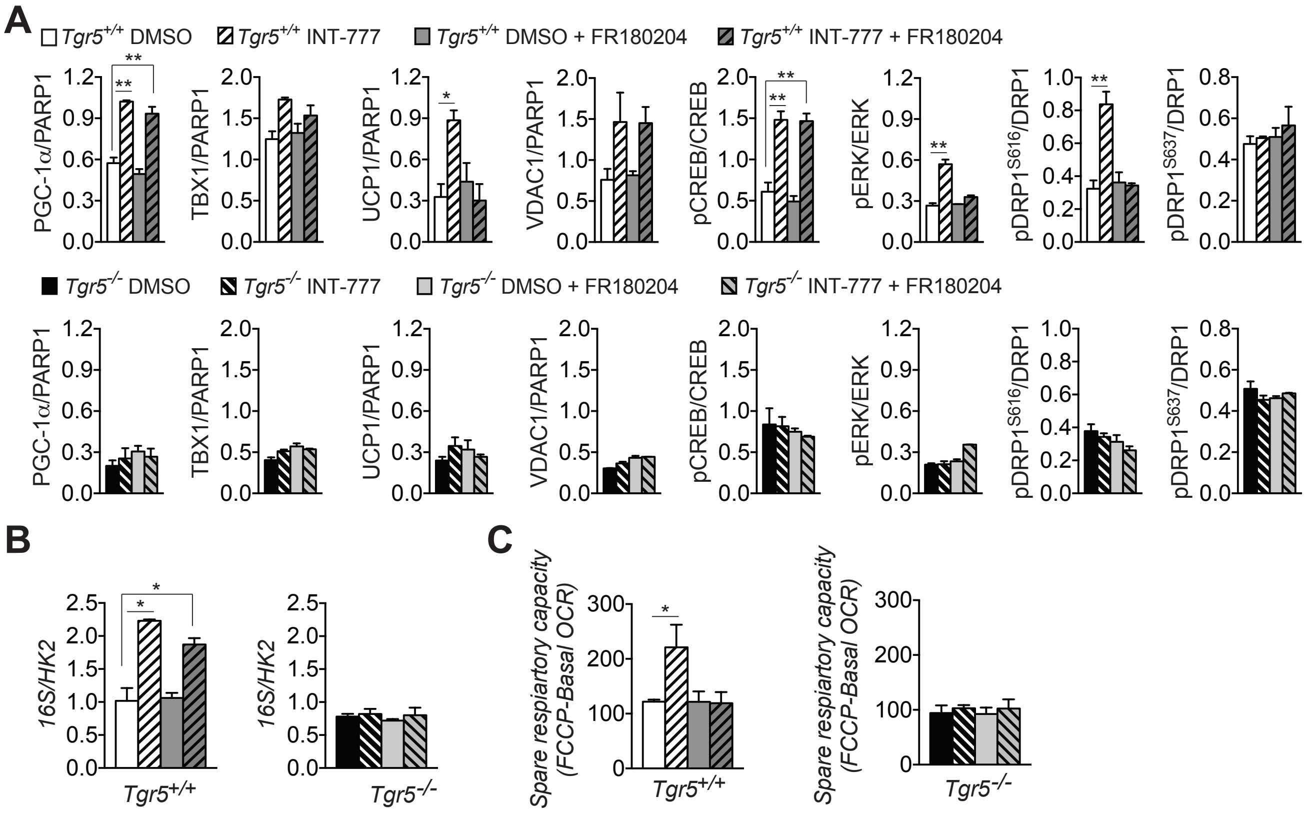

Supplementary Fig. 8. ERK activation is required for the TGR5-mediated mitochondrial fission. (**A**) Quantitative densitometry of the Western blots showed in **Figure 7, a** and **b** (*n* = 6 per group). (**B**) Quantification of mitochondrial (16S) versus nuclear (HK2) DNA ratio from differentiated adipocytes derived from the stromal vascular fraction (SVF) of TGR5 wild-type (*Tgr5*$^{+/+}$) and germline TGR5 knock-out (*Tgr5*$^{-/-}$) mice, differentiated in the presence or absence of the TGR5 agonist INT-777 and/or the ERK inhibitor FR180204. (**C**) Spare respiratory capacity calculated as the difference between maximal (FCCP-driven) and basal respiration measured in **Figure 7d** (*n* = 6 per group). Results represent mean ± SEM. * $P \leq 0.05$, ** $P \leq 0.01$ and *** $P \leq 0.001$ vs. *Tgr5*$^{+/+}$ cells by one-way ANOVA followed by Bonferroni post-hoc test.

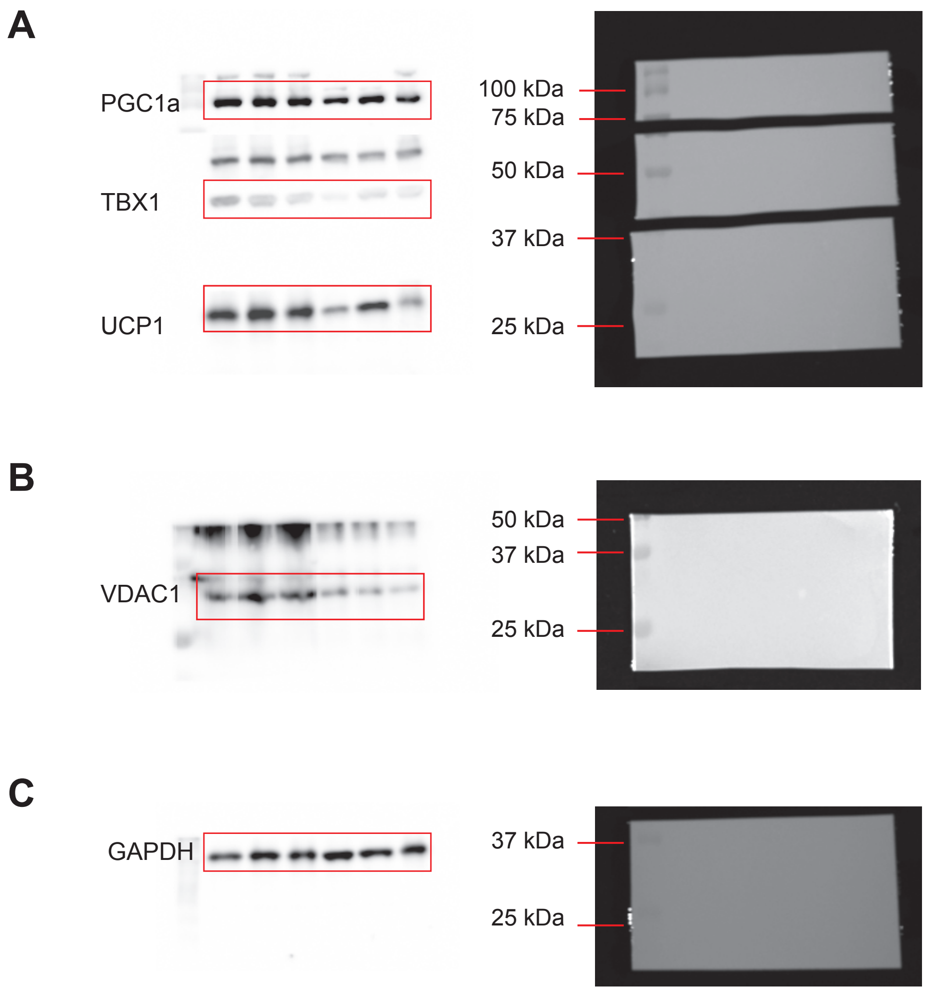

Supplementary Fig. 9. Uncropped scans of Western blots related to Figure 1. Scans of (**A**) peroxisome proliferator-activated receptor-coactivator-1 alpha (PGC1a), transcription factor T-box 1 (TBX1) and uncoupling protein 1 (UCP1), **(B)** voltage dependent anion channel 1 (VDAC1), and **(C)** glyceraldehyde 3-phosphate dehydrogenase (GAPDH) Western blots related to Figure 1f. Images represent three of more technical experiments.

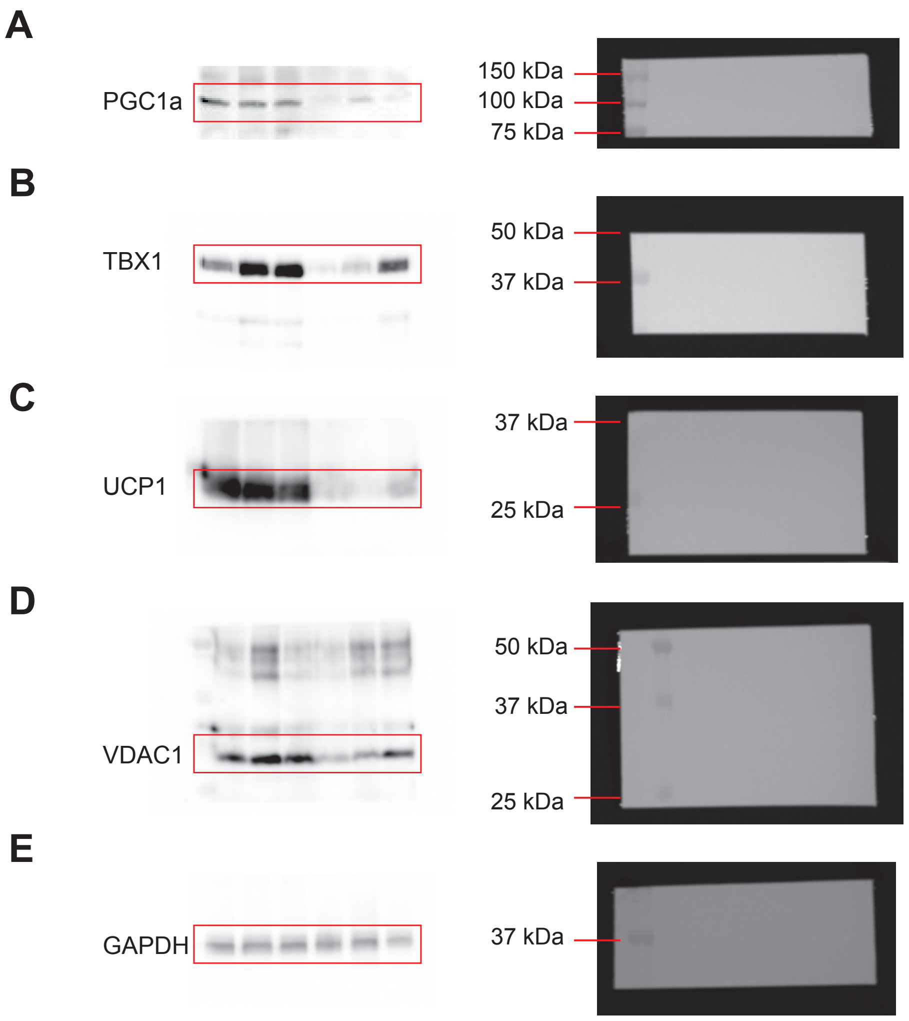

Supplementary Fig. 10. Uncropped scans of Western blots related to Figure 2. Scans of (**A**) peroxisome proliferator-activated receptor-coactivator-1 alpha (PGC1a), **(B)** transcription factor T-box 1 (TBX1), **(C)** uncoupling protein 1 (UCP1), **(D)** voltage dependent anion channel 1 (VDAC1), and **(E)** glyceraldehyde 3-phosphate dehydrogenase (GAPDH) Western blots related to Figure 2f. Images represent three of more technical experiments.

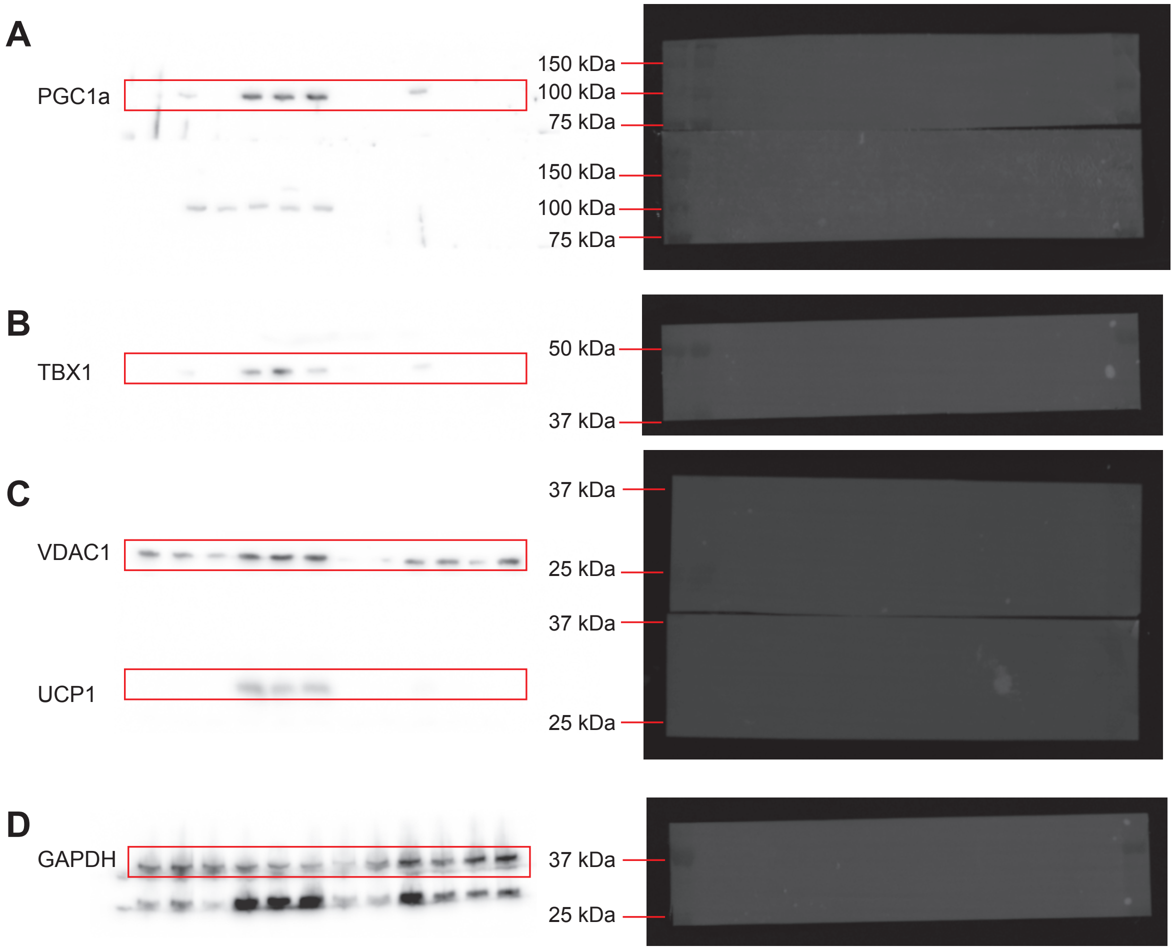

Supplementary Fig. 11. Uncropped scans of Western blots related to Figure 3. Scans of (**A**) peroxisome proliferator-activated receptor-coactivator-1 alpha (PGC1a), **(B)** transcription factor T-box 1 (TBX1), **(C)** voltage dependent anion channel 1 (VDAC1) and uncoupling protein 1 (UCP1), and **(D)** glyceraldehyde 3-phosphate dehydrogenase (GAPDH) Western blots related to Figure 3e. Images represent three of more technical experiments.

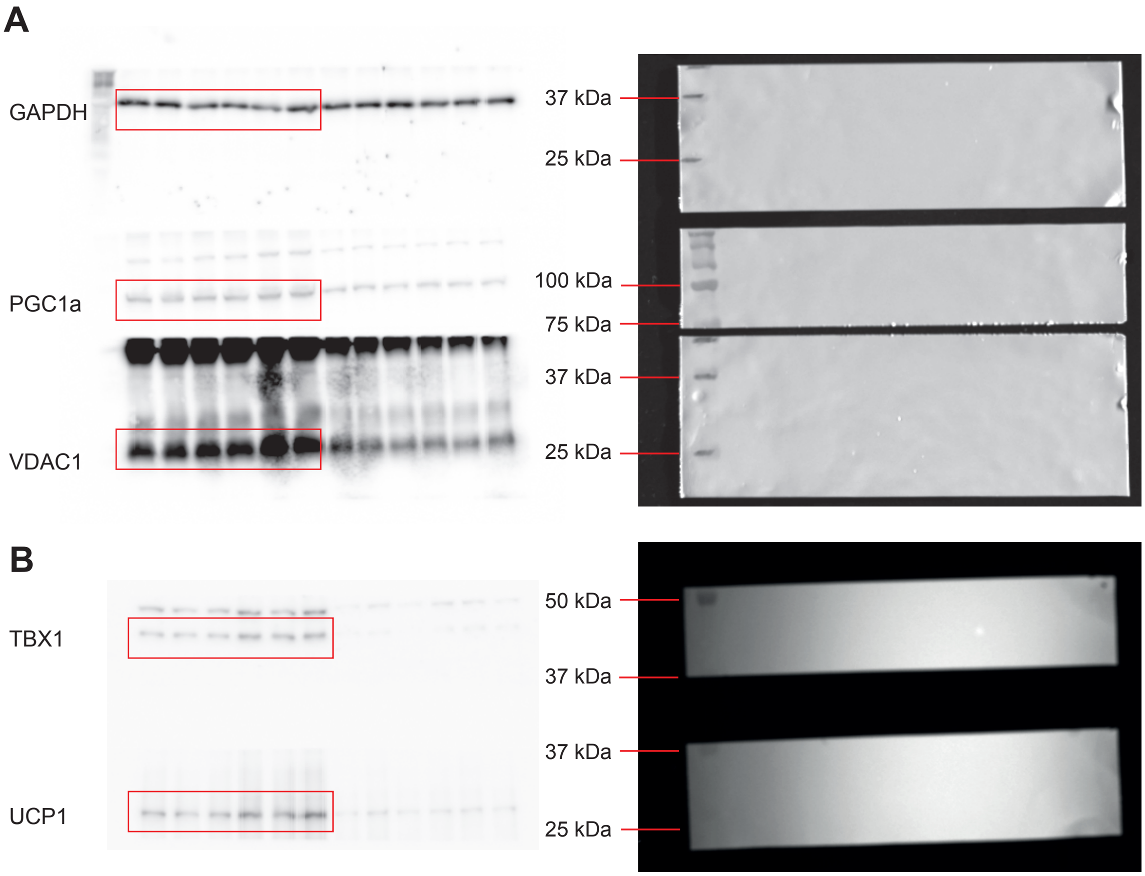

Supplementary Fig. 12. Uncropped scans of Western blots related to Figure 4. Scans of **(A)** glyceraldehyde 3-phosphate dehydrogenase (GAPDH), peroxisome proliferator-activated receptor-coactivator-1 alpha (PGC1a) and voltage dependent anion channel 1 (VDAC1), **(B)** transcription factor T-box 1 (TBX1) and uncoupling protein 1 (UCP1) Western blots related to Figure 4c. Images represent three of more technical experiments.

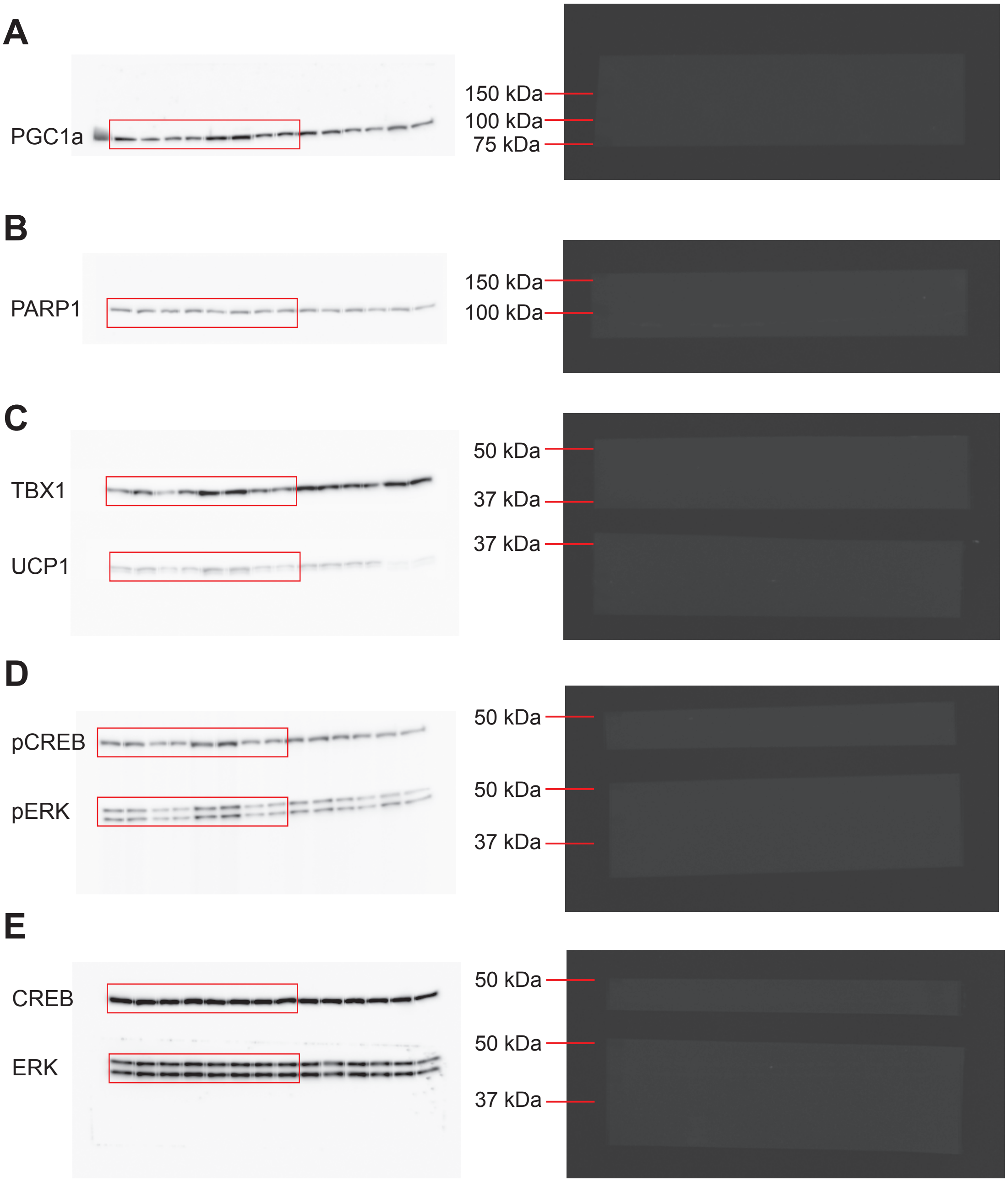

Supplementary Fig. 13. Uncropped scans of Western blots related to Figures 5 and 6. Scans of **(A)** peroxisome proliferator-activated receptor-coactivator-1 alpha (PGC1a), **(B)** poly(ADP-ribose) polymerase 1 (PARP1), **(C)** transcription factor T-box 1 (TBX1) and uncoupling protein 1 (UCP1), **(D)** phosphorylation of cyclic AMP response element binding protein (pCREB) and phosphorylation of extracellular signal-regulated kinase (pERK), and **(E)** cyclic AMP response element binding protein (CREB) and extracellular signal-regulated kinase (ERK) Western blots related to Figures 5d and 6e. Images represent three of more technical experiments.

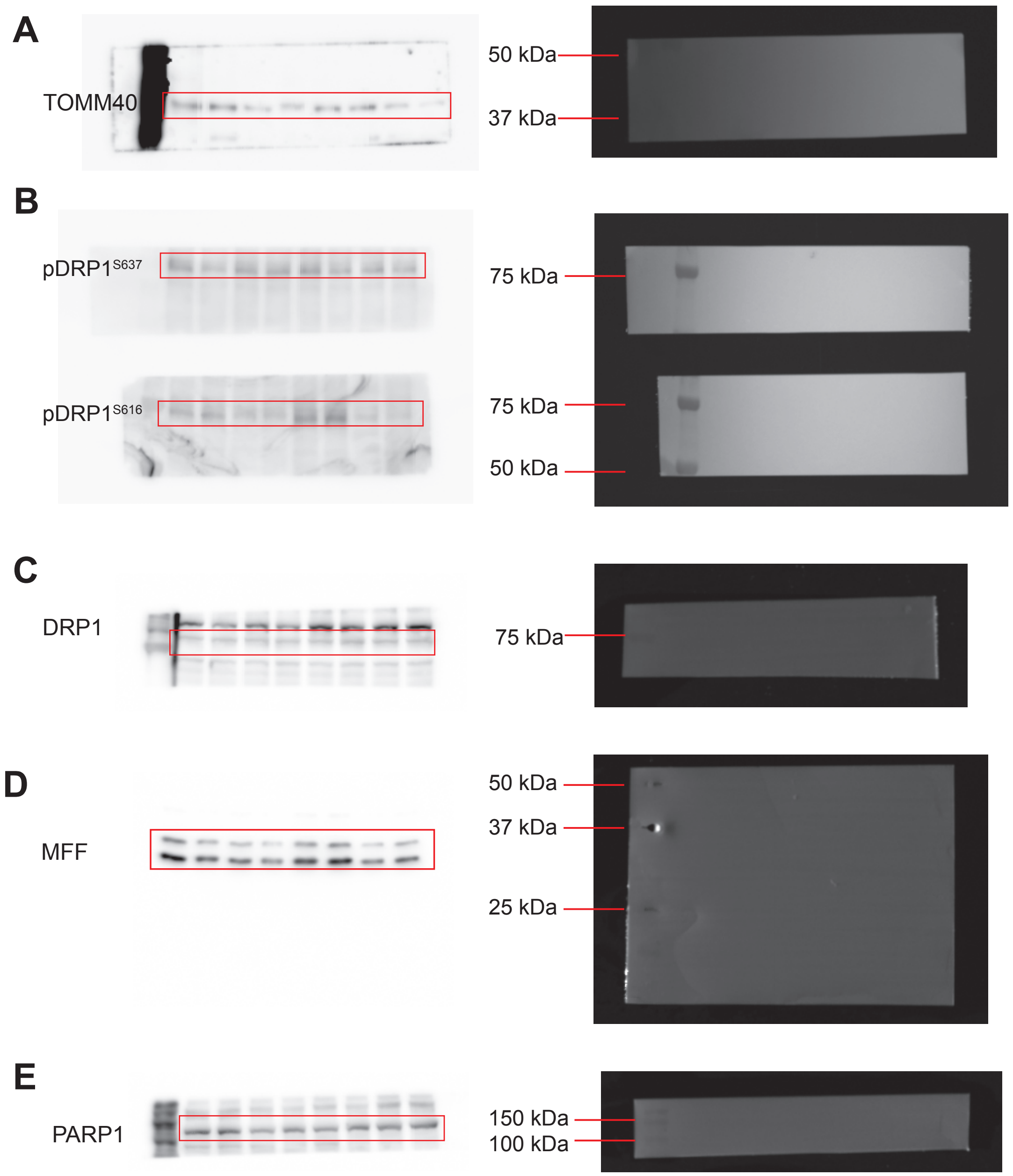

Supplementary Fig. 14. Uncropped scans of Western blots related to Figure 6. Scans of (**A**) translocase of outer mitochondrial membrane 40 (TOMM40), **(B)** phosphorylation in serine 637 of dynamin-1-like protein (pDRP1^{S637}) and phosphorylation in serine 616 of dynamin-1-like protein (pDRP1^{S616}), **(C)** dynamin-1-like protein (DRP1), **(D)** mitochondrial fission factor (MFF), and **(E)** poly(ADP-ribose) polymerase 1 (PARP1) Western blots related to Figures 5d and 6e. Images represent three of more technical experiments.

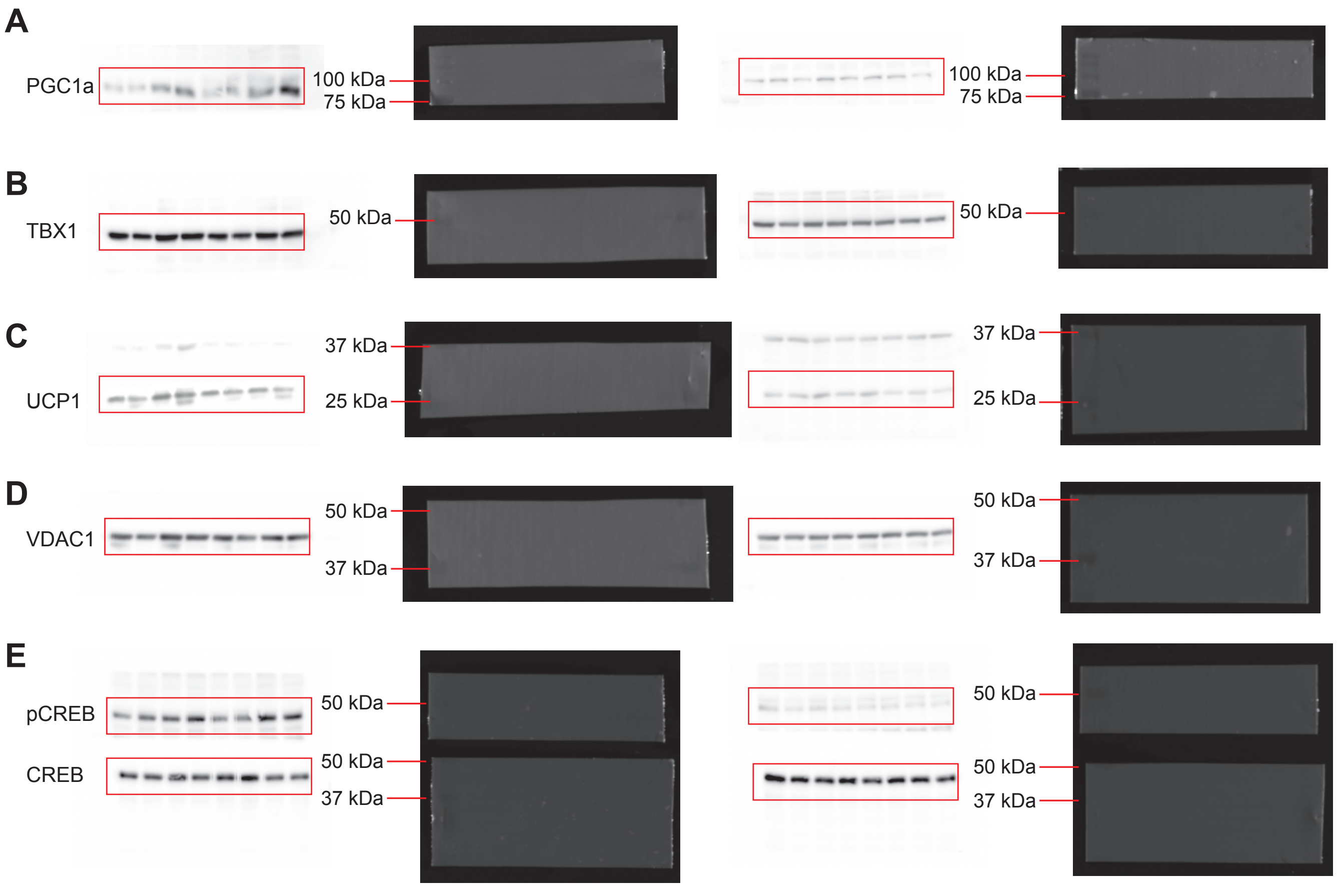

Supplementary Fig. 15. Uncropped scans of Western blots related to Figure 7 part 1. Scans of (**A**) peroxisome proliferator-activated receptor-coactivator-1 alpha (PGC1a), **(B)** transcription factor T-box 1 (TBX1), **(C)** uncoupling protein 1 (UCP1), **(D)** voltage dependent anion channel 1 (VDAC1) and **(E)** phosphorylation of cyclic AMP response element binding protein (pCREB) and cyclic AMP response element binding protein (CREB) Western blots related to Figures 7a and b. Images represent three of more technical experiments.

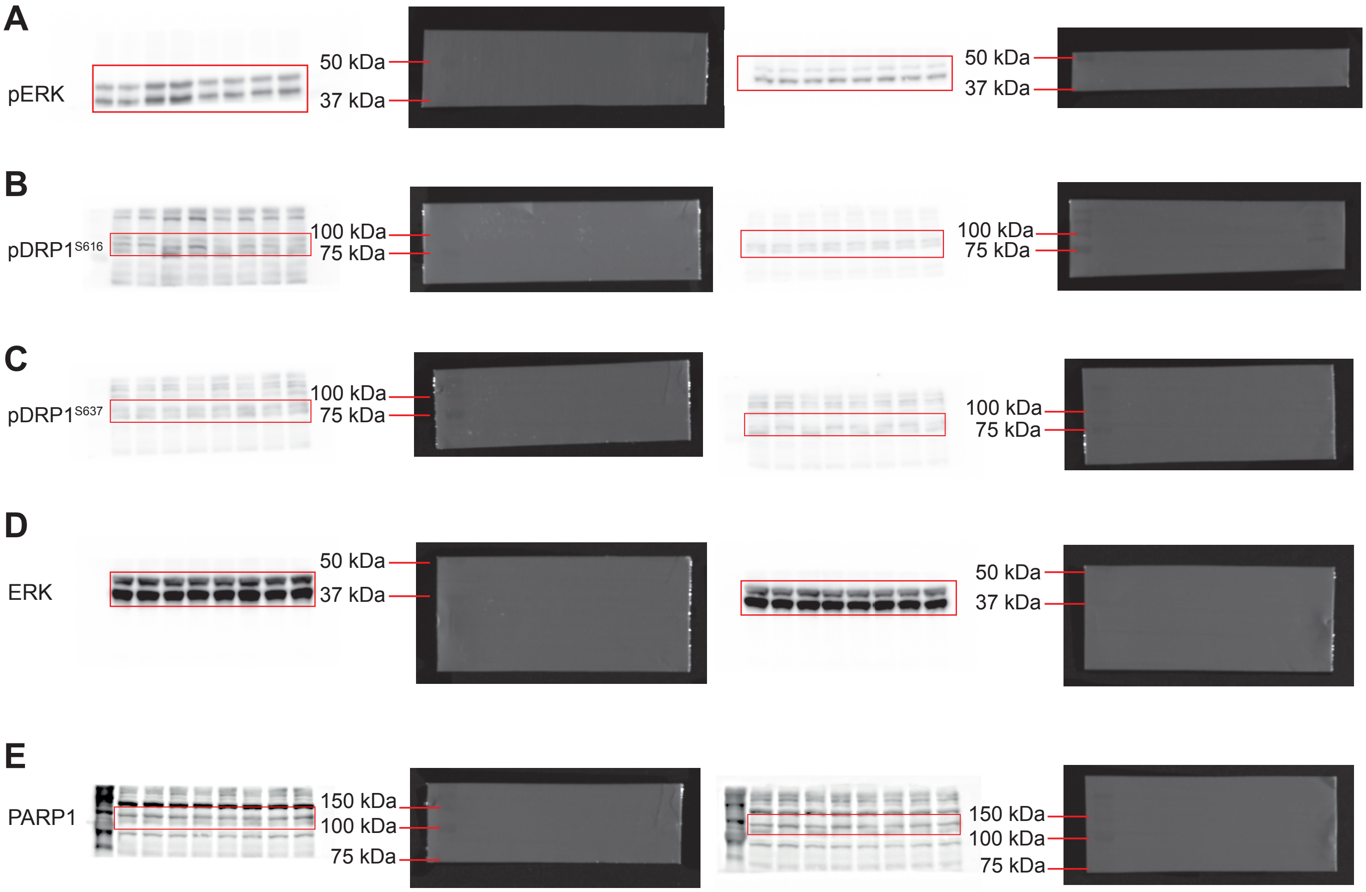

Supplementary Fig. 16. Uncropped scans of Western blots related to Figure 7 part 2. Scans of (**A**) phosphorylation of extracellular signal-regulated kinase (pERK), **(B)** phosphorylation in serine 616 of dynamin-1-like protein (pDRP1^{S616}), **(C)** phosphorylation in serine 637 of dynamin-1-like protein (pDRP1^{S637})**, (D)** extracellular signal-regulated kinase (ERK) and **(E)** poly(ADP-ribose) polymerase 1 (PARP1) Western blots related to Figures 7a and b. Images represent three of more technical experiments.

Supplementary Table 1. Primers used for qPCR

Specie	Gene	Sequence 5' – 3'
Mouse	*Pgc1a*	Fw TGAGGACCGCTAGCAAGTTT<br>Rv TGTAGCGACCAATCGGAAAT
	Ucp1	Fw CTTTGCCTCACTCAGGATTGG<br>Rv ACTGCCACACCTCCAGTCATT
	Tbx1	Fw CTGTGGGACGAGTTCAATCAG<br>Rv TTGTCATCTACGGGCACAAAG
	Prdm16	Fw CAGCACGGTGAAGCCATTC<br>Rv GCGTGCATCCGCTTGTG
	Cidea	Fw CATGATCTTGGAAAAGGGACAG<br>Rv ATCGTGGCTTTGACATTGAGAC
	Cd137	Fw CCTTGCAGGTCCTTACCTTGT<br>Rv GTTGCTTGAATATGTGGGGGA
	Pparg2	Fw TCGCTGATGCACTGCCTATG<br>Rv GAGAGGTCCACAGAGCTGATT
	Cebpb	Fw GCAAGAGCCGCGACAAG<br>Rv GGCTCGGGCAGCTGCTT
	Adrb3	Fw ACAGCAGACAGGGACAGAGG<br>Rv TCCTGTCTTGACACTCCCTCA
	Th	Fw GTCTCAGAGCAGGATACCAAGC<br>Rv CTCTCCTCGAATACCACAGCC
	Tgr5	Fw TCCTGTCAGTCTTGGCCTATGA<br>Rv GGTGCTGCCCAATGAGATG
	36b4	Fw AGATTCGGGATATGCTGTTGG<br>Rv AAAGCCTGGAAGAAGGAGGTC
	B2m	Fw CTGACGAAGGCCACTTTGCACCTCTG<br>Rv GCACAAGATCCCGGTCAAGTACCTGGAG
	16S	Fw CCGCAAGGGAAAGATGAAAGAC<br>Rv TCGTTTGGTTTCGGGGTTTC
	Hk2	Fw GCCAGCCTCTCCTGATTTTAGTGT<br>Rv GGGAACACAAAAGACCTCTTCTGG
Human	*Pgc1a*	Fw TCTGAGTCTGTATGGAGTGACAT<br>Rv CCAAGTCGTTCACATCTAGTTCA
	Ucp1	Fw AGGTCCAAGGTGAATGCCC<br>Rv GCGGTGATTGTTCCCAGGA
	Tbx1	Fw TAGCGAGAAATATGCCGAGGA<br>Rv CGTGATCCGATGGTTCTGGT
	Prdm16	Fw CGAGGCCCCTGTCTACATTC<br>Rv GCTCCCATCCGAAGTCTGTC
	Cidea	Fw TTATGGGATCACAGACTAAGCGA<br>Rv TGCTCCTGTCATGGTTGGAGA
	Cd137	Fw AGCTGTTACAACATAGTAGCCAC<br>Rv TCCTGCAATGATCTTGTCCTCT
	Pparg2	Fw CAGCAAACCCCTATTCCATGC<br>Rv TCTCCGGAAGAAACCCTTGC
	Cebpb	Fw AGAGCAAGGCCAAGAAGACC<br>Rv GACAGCTGCTCCACCTTCTT
	Hprt	Fw TGGGATTACACGTGTGAACCAACC<br>Rv GCTCTACCCTCTCCTCTACCGTCC
